# Brain Oscillations in Sport: Toward EEG Biomarkers of Performance

**DOI:** 10.3389/fpsyg.2016.00246

**Published:** 2016-02-26

**Authors:** Guy Cheron, Géraldine Petit, Julian Cheron, Axelle Leroy, Anita Cebolla, Carlos Cevallos, Mathieu Petieau, Thomas Hoellinger, David Zarka, Anne-Marie Clarinval, Bernard Dan

**Affiliations:** ^1^Laboratory of Neurophysiology and Movement Biomechanics, Université Libre de Bruxelles Neuroscience Institut, Université Libre de BruxellesBrussels, Belgium; ^2^Laboratory of Electrophysiology, Université de Mons-HainautMons, Belgium; ^3^Haute Ecole CondorcetCharleroi, Belgium; ^4^Inkendaal Rehabilitation HospitalVlezembeek, Belgium

**Keywords:** EEG, biomarkers, sport, brain rythms, cortical activity

## Abstract

Brain dynamics is at the basis of top performance accomplishment in sports. The search for neural biomarkers of performance remains a challenge in movement science and sport psychology. The non-invasive nature of high-density electroencephalography (EEG) recording has made it a most promising avenue for providing quantitative feedback to practitioners and coaches. Here, we review the current relevance of the main types of EEG oscillations in order to trace a perspective for future practical applications of EEG and event-related potentials (ERP) in sport. In this context, the hypotheses of unified brain rhythms and continuity between wake and sleep states should provide a functional template for EEG biomarkers in sport. The oscillations in the thalamo-cortical and hippocampal circuitry including the physiology of the place cells and the grid cells provide a frame of reference for the analysis of delta, theta, beta, alpha (incl.mu), and gamma oscillations recorded in the space field of human performance. Based on recent neuronal models facilitating the distinction between the different dynamic regimes (selective gating and binding) in these different oscillations we suggest an integrated approach articulating together the classical biomechanical factors (3D movements and EMG) and the high-density EEG and ERP signals to allow finer mathematical analysis to optimize sport performance, such as microstates, coherency/directionality analysis and neural generators.

## Introduction

For top performance accomplishment in sports, the brain dynamics needs to be taken into account because it determines both motor control and crucial psychological factors, such as intrinsic motivation (Pedersen, 2002), selective attention (Arjona et al., 2014; Abdollahipour et al., 2015), goal setting (West and Thorn, 2001), working memory (Dipoppa and Gutkin, 2013), decision making (Renfree et al., 2014), positive self-concept (Badami et al., 2012), and self-control (Ali et al., 2012; Chiviacowsky et al., 2012).

**Figure 1:**
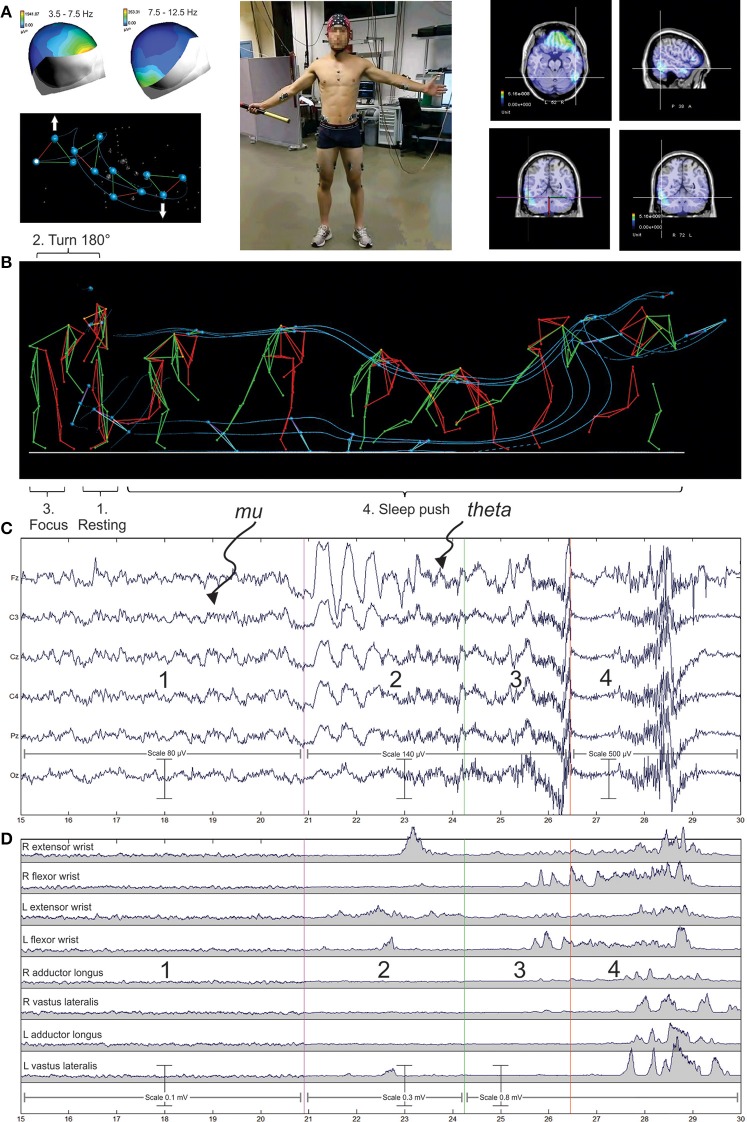
**Illustration of an integrated neurophysiological approach in sport**. The sleep push or drag flicking in field hockey sport is here illustrated as an example of complex movement involving the whole body. It is an attacking movement mainly performed within the penalty corner but also realized outside the penalty corner situation. Here in our experimental setup, the player was placed in front of the laboratory wall with the field of action in its back. He was asked to relax in this position before turning in front of the action field. Then he performed the sleep push movement at its self-paced mode in order to put the ball inside of the goal. **(A)** The player was equipped with a 128 electrodes cap (ANT), the cables of the cap were connected to the amplifier which were placed in a traveling box piloted by the experimenter in order to assume the follow-up of the player's running. **(B)** Surfaces electrodes () were placed on muscles of interest and allowed the electromyographic (EMG) recording (see, G). **(C)** Retro-reflective passive markers were fixed on the skin overlying the following bony landmarks on both sides of the body: LM, lateral malleolus; MT, fifth metatarsal head; LE, lateral condyle of the knee; GT, greater trochanter; and IL, tubercule of the anterosuperior iliac crest then a stick diagram of the whole body representation was constructed. **(D)** Kinematics of the sleep push movement, successive pictures of the main phase are presented and 3 markers placed on the head and 3 other placed on the distal part of the stick are joined by continuous lines (blue) giving a global representation of the movement signature.

In practical terms, neuronal oscillations can be readily recorded in a non-invasive way in human allowing the possibility to follow the dynamics of brain working during activity. The optimized state of performance reached by sport elites offers a privileged domain for studying the different neuronal oscillations linked to sensorimotor and cognitive control getting to final success or failure. For example, Figure 1 illustrates the recent integrated approach performed by our team in elite hockey players during the accomplishment of the sleep push movement. The sleep push involves a player crouching low down, picking the ball up on the shaft of the stick, then moving in a forceful slinging action of the ball around the running up body. This allows accelerating the ball in the direction of the goal. Such approach allows establishing functional links between the multiple neurophysiological signals in order to identify the different neural generators in the brain (Figure 1).

The aim of such studies is the characterization of an array of electroencephalographic (EEG) dynamics biomarkers of sport performance analogous to those proposed in mental health (Başar et al., 2013; Olbrich and Arns, 2013; Loo et al., 2015). However, our current understanding of the neuronal oscillations that occur before, during and after a dedicated sport movement remains problematic because of the absence of consensus about physiological significance of the different oscillatory signals recorded from the scalp, the absence of standardized methodologies for sport domain, and the presence of different artifacts due to sport movement itself (Thompson et al., 2008; Reinecke et al., 2011; Castermans et al., 2014; Kline et al., 2015). In addition, useful biomarkers will need to integrate neuronal models of flexibility such as the one elaborated for working memory process (Dipoppa and Gutkin, 2013, see below). Indeed, the different types of cortical oscillations must not be understood separately but in close interaction in which subtle physiological mechanisms operate.

In this inaugural article we intent to review the current relevance of the main types of EEG oscillations in order to trace a perspective for future practical applications in sport domain.

## The EEG origin and information content

Thanks to its non-invasive nature, EEG recording has been recognized as one of the most routinely neuroimaging approach for studying brain activities (Niedermeyer and da Silva, 1982). Nevertheless, it remains unknown which part of the complex neurophysiological events occurring in the cortical mantle are represented by the surface EEG, in spite of their clinical interest (Libenson, 2012) and numerous applications in psychology (Dickter and Kieffaber, 2014) and in the emergent domains of sport science (Baumeister et al., 2008; Park et al., 2015) and brain computing interface (BCI) (Cheron et al., 2009; Duvinage et al., 2012; Angulo-Sherman and Gutiérrez, 2015). Therefore, in this review, we shall critically revisit the different EEG rhythms which might form a template for future biomarkers of performance.

### Neuronal membrane potentials and neuronal synchrony

**Figure 2:**
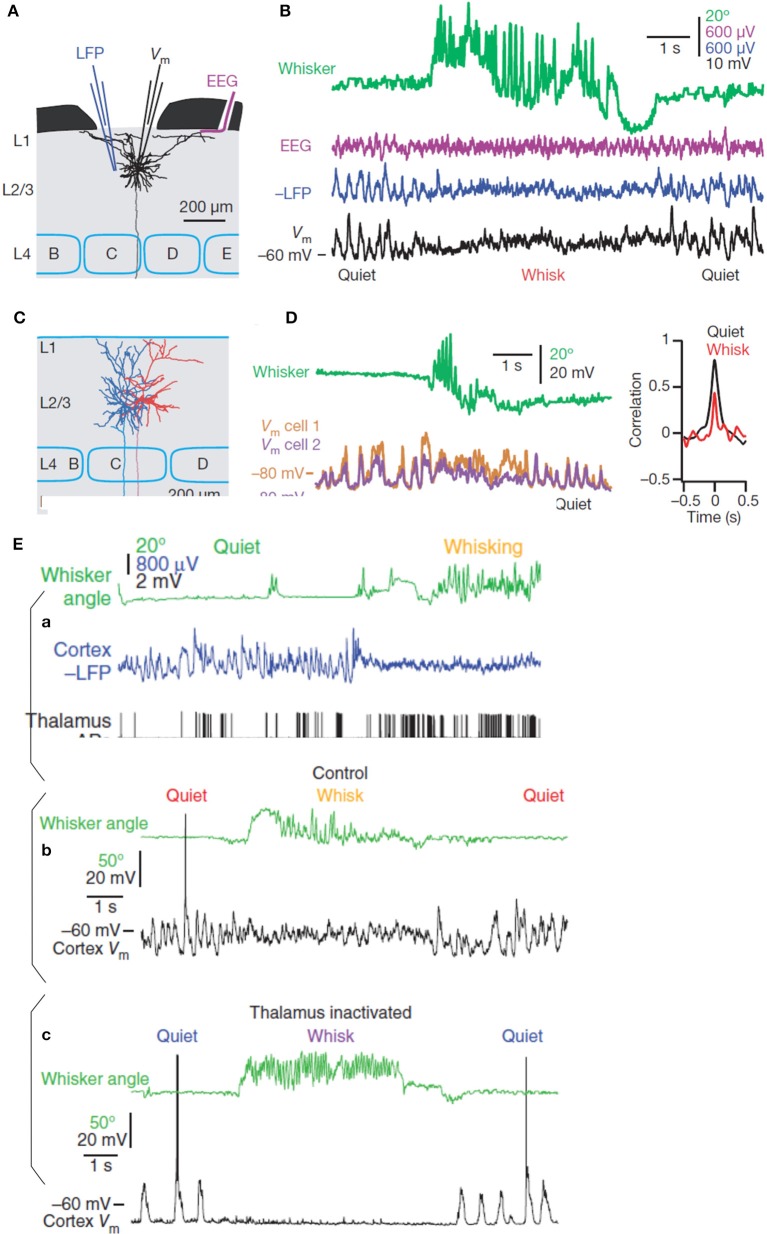
**(A)** Recording configuration (simultaneous EEG, LFP, and whole-cell recordings) in the mouse somatosensory cortex (Barrel cortex) and reconstruction of one excitatory pyramidal neuron (black) of the layer 2/3. **(B)** From top to bottom, movement of the whisker (green) defining quiet and whisking periods, EEG signals (red), LFP recording (blue), and membrane potentials (black). **(C)** Reconstruction of two layer 2/3 pyramidal neurons (blue and red) simultaneously recorded. **(D)** From top to bottom, whisking movement (green) and superimposition of the membrane potentials of the two pyramidal neurons with their cross-correlation during quiet and whisking movement (modified from Poulet and Petersen, 2008, with permission). **(E)** Simultaneous recording of a thalamic neuron and cortical LFP showing the increase of the action potential firing rates during the active desynchronized cortical state **(a)**. Behavioral difference of the membrane potential of a pyramidal neuron in presence **(b)**, or in absence of the thalamic influence **(c)** during quiet and whisking states (see text for more details, modified from Poulet et al., 2012, with permission).

The recent approaches coupling electrocorticographic (EcoG), local field potentials (LFP) and dual whole-cell recording in the primary somatosensory barrel cortex in behaving mice (Figure 2) (Poulet and Petersen, 2008) have revealed interesting results about the contribution of the membrane potential (V(m)) and the neuronal synchrony to EEG recording. They demonstrated a perfect synchrony between the V(m) of a large number of pyramidal cell pairs in such a way that LFP and EEG signals represent a close reflection of the rhythmic fluctuation of the synchronized V(m) highly correlated in the resting state and less correlated during movement (Figure 2D). The desynchronized rhythmicity during movement can be due to the influence of afferent signals or to central reverberating activity. In fact, the two oscillatory states (resting and active) are not dependent on afferent information (Poulet and Petersen, 2008). These cortical states are centrally produced and controlled by the thalamus independently from afferent and efferent signals (Figure 2E; Poulet et al., 2012). Interestingly, the inhibitory interneurons of the somatosensory cortex fire at significantly higher frequencies than excitatory neurons during the resting state (Gentet et al., 2010).

Although whisking movement of mouse is not directly related to sport movement in human these results shed a new light on the mechanism underlying the event related spectral perturbation (ERSP) analysis applied on the EEG recorded in sport. In particular, it highlights the significance of event related desynchronization (ERD) and event related synchronization (ERS) episodes of the EEG signals with respect to selected inhibitory or excitatory neuronal populations.

## Unified brain rhythms and continuity hypothesis between wake and sleep states

Self-emerging oscillation occupy a central position because they represent the brain's fundamental organizer of neuronal information (Varela et al., 2001; Buzsáki, 2006; Buzsáki and Watson, 2012; Başar et al., 2013; Cheron et al., 2015). Neuronal oscillators are implicated in a plethora of crucial functions extending from movement control and cognition to sleep. In accordance with the concept of unified brain rhythms, the continuity hypothesis (Pesant and Zadra, 2006; Marzano et al., 2011; Fullagar et al., 2015) proposed that there is a sort of permanency of encoding and retrieval mechanisms of episodic memories across sleep and wakefulness (Scarpelli et al., 2015). Based on these evidences, we suggest that there is continuum of brain oscillatory flux ranging from slow to fast rhythms throughout the day and night that must be taken into account and practically applied in the follow-up of athletes (Fullagar et al., 2015).

### The quality of sleep oscillations in sport: slow-wave oscillation (~1 Hz)

Sport practice has multiple impacts on brain-body relationships, including during following night and day after. This implies that the follow-up of the EEG should also consider the quality of sleep oscillations.

**Figure 3:**
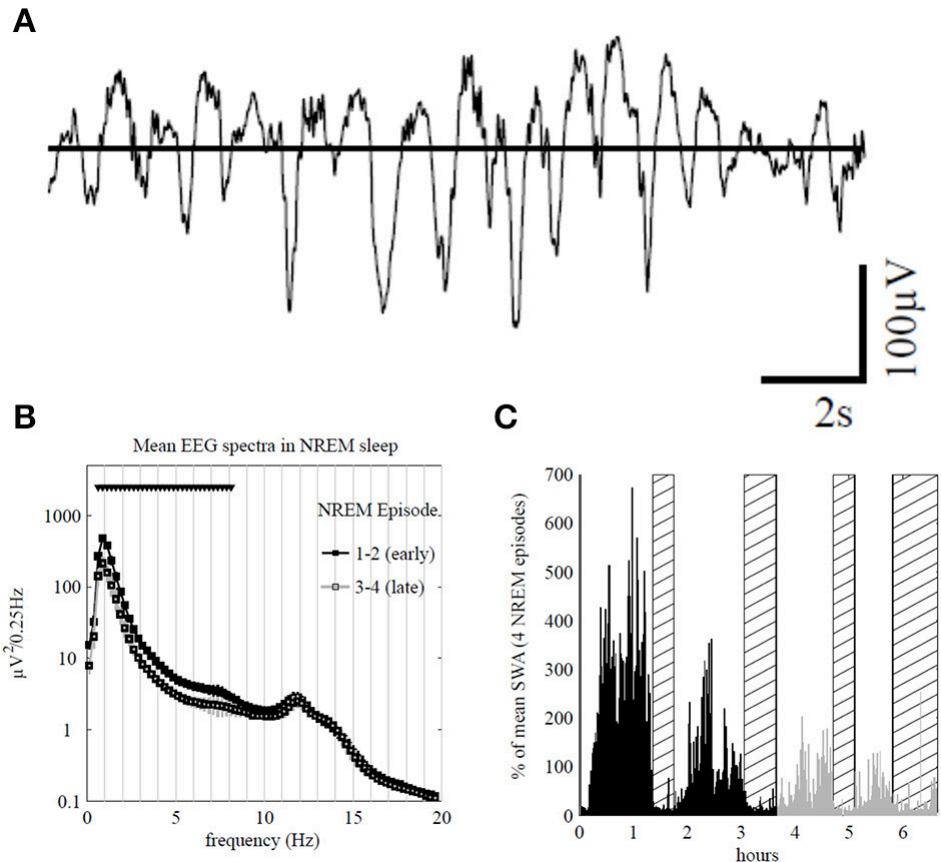
**(A)** Top traces: representative 16 s electroencephalogram (EEG) traces from the Fp1 channel during early sleep. **(B)** Average power spectra in non-rapid eye movement (NREM) sleep during episodes 1 and 2 (early sleep, black) and episodes 3 and 4 (late sleep, gray) for Fp1 channel (mean ± SEM, *n* = 7). Triangles indicate significant bins based on SnPM (*P* < 0.05, single threshold corrected). **(C)** Slow-wave activity (SWA; 0.5–4.0 Hz) profile in NREM sleep during the night for an individual subject (average 1-min values, % of the mean of 4 NREM episodes) rapid eye movement (REM) episodes are indicated by hatched areas. Early and late sleep (including REM episodes) are color-coded in black and gray, respectively. Note that 69% of the sleep time corresponds to NREM, 23% of REM and the rest of time refers to waking after sleep onset. In addition, about 17% of the NREM correspond to the SWS (adapted from Riedner et al., 2007, with permission).

For example, the loss of the slow-wave activity (SWA) occurring during the non-rapid eye movement (NREM) state (Figure 3) is related to slower EEG and impaired performance due to cognitive and memory deficits during wake (Bellesi et al., 2014). The amount of SWA is homeostatically regulated; SWA increase after a learning task concerning specific brain areas is accompanied by improved performance after sleep (Huber et al., 2004). In line with this local plasticity exerted by the SWA it was demonstrated that the immobilization of the subject's arm during the day was accompanied by local SWA reduction during the subsequent sleep (Huber et al., 2006). This is indicative of a local synaptic depression linked to somatosensory and motor evoked potentials in the contralateral sensorimotor cortex and a deterioration of the motor performance.

### Fast sleep spindles (13–15 Hz) and new motor sequences

It is interesting to note that in spite of the fact that the 1 Hz oscillation largely dominates the EEG frequency spectrum, a clear alpha peak (~12 Hz) corresponding to sleep spindle activity remains stable during all the NREM episodes (Figure 3B). The studies related to the effects of sleep spindles on motor performance showed that fast spindles (13–15 Hz) and not the slow spindles (11–13 Hz) were favorable for the consolidation of new motor sequences (Barakat et al., 2011; Astill et al., 2014). Another example of local cortical plasticity and sleep oscillations was given by the effect of a long-term synaptic potentiation (LTP) produced with transcranial magnetic stimulation (TMS) over the contralateral motor cortex (De Gennaro et al., 2008). Performed during waking, this LTP affected delta and theta EEG power in both NREM and REM states, recorded during the following night, in such a way that SWA was increased in frontal and prefrontal cortex and decreased in the contralateral sites of the stimulated motor cortex (De Gennaro et al., 2008). Similar relationships between SWA and LTP were demonstrated in long-term synaptic depression (LTD) (Bergmann et al., 2008). These experimental evidences corroborate the synaptic homeostasis hypothesis of Tononi and Cirelli (2014), who suggested that sleep is *the price the brain pays for plasticity*. According to this hypothesis, the oscillatory activity of normal sleep allows the brain to reestablish synaptic and cellular homeostasis which has been confronted to environmental demands implicating energetic supplies and plastic changes occurring during wake. In the same vein, the restorative function of sleep also consists of removing potentially neurotoxic waste products that accumulate in relation to awake brain activity (Xie et al., 2013).

### The sharp wave-ripple (SPW-R)

**Figure 4:**
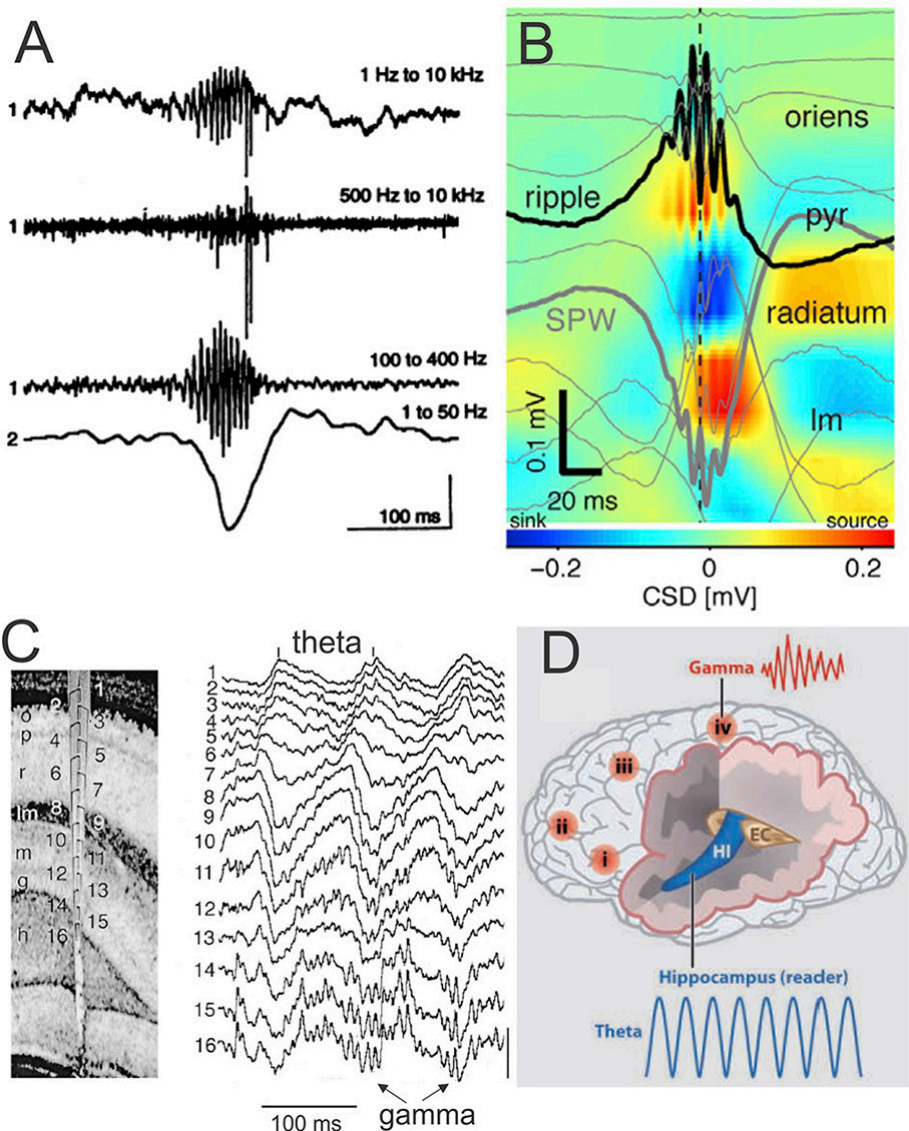
**SPW-R is composed by a large and sharp amplitude depolarization (SPW) of about 40-100 ms recorded in the hippocampus CA1 stratum radiatum and most often associated by brief and fast LFP oscillation (~110–200 Hz) known as “ripples” (R) in the CA1 pyramidal layer**. The SPW-R represents one of the most synchronous network patterns implicating synchronicity of 10–20% of hippocampus neurons (Chrobak and Buzsáki, 1994). **(A)** Fast field oscillation in the CA1 region of the dorsal hippocampus. Simultaneous recordings from the CA1 pyramidal layer [electrode 1, wide-band recording (1 Hz–10 kHz)] and stratum radiatum (electrode 2, unit activity 500 Hz–10 kHz and fast field oscillation (100–400 Hz). The second and third traces are digitally filtered derivatives of the wide-band trace (1). Note simultaneous occurrence of fast field oscillations, unit discharges, and sharp amplitude depolarization (electrode 2). Calibrations: 0.5 mV (trace 1), 0.25 mV (traces 2 and 3), and 1.0 mV (trace 4) (from Buzsáki et al., 1992, with permission). **(B)** Depth profile of averaged sharp-wave ripples (SPW-R) in a freely moving mouse (*n* = 961 events; vertical site separation: 100 mm). Voltage traces (light gray) are superimposed on current-source density (CSD) map. Black trace: site of maximum amplitude ripple; heavy gray trace: site of maximum amplitude SPW (adapted from Stark et al., 2014, with permission). **(C)** Depth Profile of theta and gamma oscillations in the rat recorded during exploration (left) with a 16-site silicon probe introduced in the CA1-dentate gyrus axis. Numbers indicate recording sites (100 μm spacing). Note the gradual shift of theta phase from str. oriens to str. lacunosum-moleculare (right) and the emergence of gamma waves superimposed on theta oscillation mainly in the granule cell layer and the hilus. Vertical bar: 1 mV (from Bragin et al., 1995, with permission). **(D)** Schematic representation of the brain showing location of γ oscillations in different cortical areas (i–iv) and Θ oscillation in the hippocampus (HI) entorhinal cortex (EC). These brain oscillations can influence each other within and across networks by modulating the phase and/or the amplitude of the oscillations (modified from Buzsáki and Watson, 2012, with permission). Abbreviations: p or pyr, pyramidal layer; lm, str. lacunosum-moleculare; o, str. oriens; r, str. radiatum; lm, str. lacunosum-moleculare; g, granule cell layer; h, hilus.

The SPW-R is another crucial oscillation in the memory consolidation process (see details in Figure 4; O'Keefe, 1976; Buzsáki et al., 1992; Ylinen et al., 1995; Buzsáki, 2006). Generated by an intrinsic self-organizing process assuming the mechanism of information transfer from the hippocampus to neocortex during off-line states such as NREM sleep (Buzsáki and Watson, 2012; Watson and Buzsáki, 2015), the SPW-R is considered to link pre-existing and recently acquired information in order to influence future decisions and actions favoring creativity (Buzsáki, 2015).

### Viscerosensory brain, emotion, and theta oscillation during REM sleep

In accordance to functional continuity between sleep and wake state, and its role in memory consolidation (Nishida et al., 2009), theta oscillation may be detected during REM-sleep (Marzano et al., 2011) Various aspects of memory processes have been the focus of increasing interest in sports. The viscerosensory brain activation linking central and peripheral emotion-signaling systems suggests a role for REM sleep in affective brain recalibration as recently demonstrated by testing the social threat discrimination (Goldstein-Piekarski et al., 2015). The influence of emotion in sport has been well-demonstrated (Lane et al., 2011; Uphill et al., 2014). For exemple, better physical performances occurred when the athlete recall anger or happiness emotions in contrast to the emotion-neutral state (Rathschlag and Memmert, 2013). Pleasant (as opposed to unpleasant) emotion is associated with an increase in frontal midline theta power (Sammler et al., 2007).

### Prefrontal cortex and sport

Interestingly, it was demonstrated that cathodal transcranial direct current stimulation (tDCS) applied over the left dorsolateral prefrontal cotex (PFC) facilitates implicit motor learning in a golf putting task (Zhu et al., 2015). Running enhances cognitive tasks performance associated to astrocytic and neuronal plasticity in the PFC (Brockett et al., 2015). These elements may suppose an important role played by the relative hypoactivation of the PFC during dreaming (Maquet et al., 1996; Hobson et al., 2000) and call for further examination of PFC oscillations the days and nights before sport competition.

## The place cells and the grid cells: a basic template for navigation in sport

**Figure 5:**
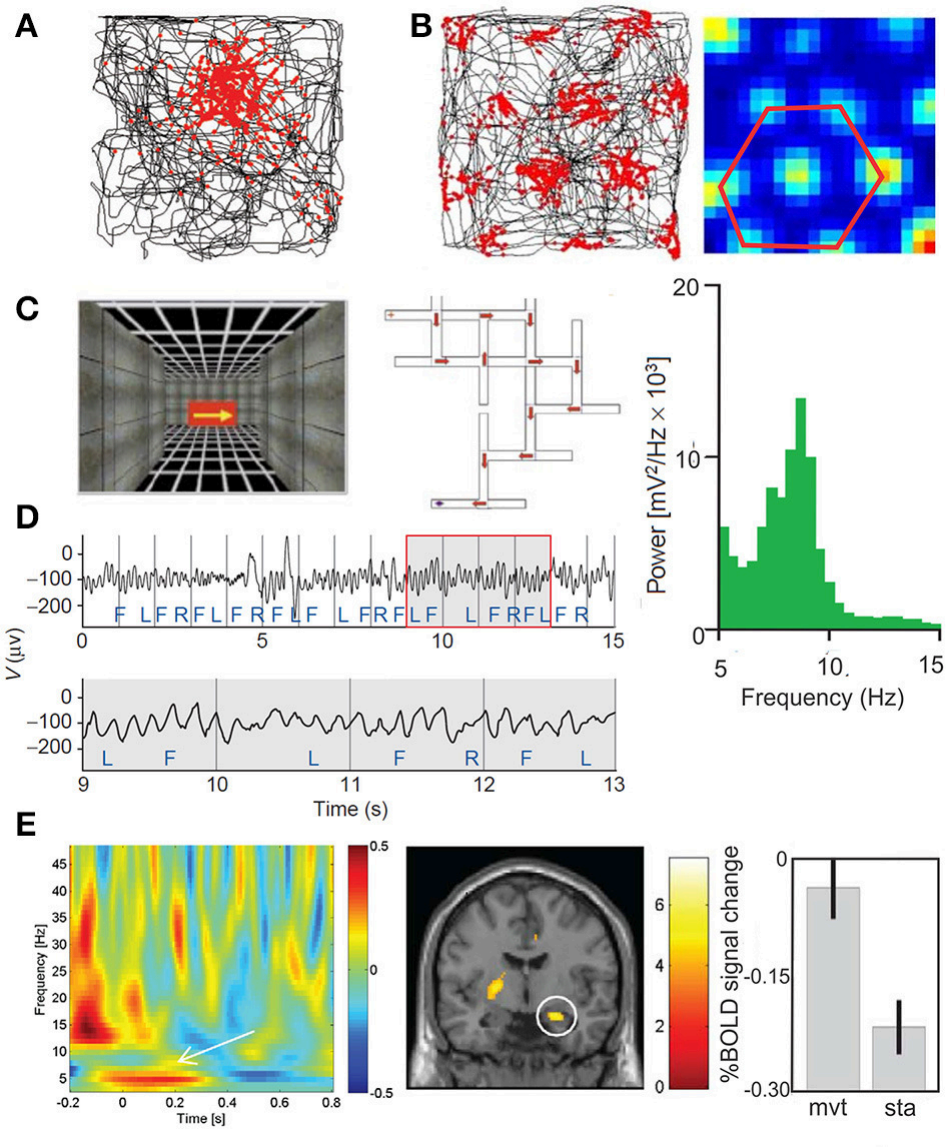
**(A)** Place cell in the hippocampus (adapted from Moser et al., 2008, with permission) and **(B)** grid cell in the medial entorhinal cortex. The locations where the neurons discharge (red) are superimposed on the rat's trajectory (black) in the recording enclosure black. Whereas, most of the place cells have a single firing location, the of a grid cell form a periodic triangular matrix of the firing fields covering the entire environment available to the animal. **(C–E)** Theta oscillations revealed by intracranial EEG and MEG in the human. **(C)** Navigation in virtual reality-rendered multiple T-junction mazes in which key presses move the subject in the VR maze (F, forward; R, right; and L, left). **(D)** Sample intracranial EEG recorded in the inferior frontal gyrus (upper trace); theta oscillation is emphasizing in an enlarged view of the boxed region (lower trace). The average power in iEEG for one trial through the VR maze during 21 s is represented by the power frequency plot (green) showing a peak in the theta band (4–12 Hz) (adapted from Kahana et al., 1999, with permission). **(E)** Movement-related MEG time-frequency effects and related fMRI effects. Plots show MEG signal as baseline corrected log normalized difference scores (dB, z axis) against time (x-axis, seconds) and frequency (y-axis, Hz), averaged across 18 participants. The effect of movement initiation during navigation (left panel) is showed at an exemplary single sensor of interest, (posterior right middle temporal location). Coronal slices showing right hippocampal activation for movement initiation compared to stationary periods (middle panel). Percent signal change at right hippocampal peak-voxel averaged across 14 participants for movement and stationary periods (mean 6 SEM) (right panel) (adapted from Kaplan et al., 2012, with permission).

The different hippocampal oscillations are highly relevant for sport performance. In addition to its crucial role in the consolidation of declarative memory (Squire, 1992), the hippocampus and related network are crucial for navigation. Indeed, place cells in the hippocampus (O'Keefe, 1976), head-direction cells in the anterior thalamic nucleus (Stackman and Taube, 1997; Giocomo et al., 2014; Winter et al., 2015), border cells (Solstad et al., 2008), boundary vector cells (Lever et al., 2009; Stewart et al., 2014) and grid cells in the medial entorhinal cortex (MEC) (Hafting et al., 2005; Fyhn et al., 2008; Moser et al., 2008; Ray et al., 2014) must be considered as the most basic elements supporting strategic navigation and self-positional control of the body. They may be thus translated for any action in the space field of human performance (Figure 5).

Following the influence of the oscillatory dynamics of the hippocampus and entorhinal cortex in memory (Squire, 1992) and spatialization (O'Keefe and Burgess, 2005; Buzsáki and Moser, 2013), navigation in a real physical world or in mental space might use similar neuronal processing and might be consequently trained during mental motor imagery tasks (de Lange et al., 2008; Cevallos et al., 2015) or during VR observation (Zarka et al., 2014) or navigation specifically dedicated to sport performance or to neurological rehabilitation and prevention.

## Delta oscillation

Delta-band oscillations have long been believed to reflect periods of deep sleep (Figure 3; Steriade, 2006). However, they have recently been shown to be both stimulus informative (Montemurro et al., 2008) and linked to attentional selection (Lakatos et al., 2008). Following Whittingstall and Logothetis (2009), delta oscillation might represent the cyclical variations in the excitability of the neuronal pool represented by multiple unit activity (MUA) and the network state and might amplify (in case of high-excitability phase) or suppress (in case of low-excitability phase) the input signals. Interestingly, this also corroborates human MEG recordings where the success in visual discrimination task depended on effective coupling between delta oscillation (1–5 Hz) and high-frequency oscillations in the gamma band (~ 63 Hz) (Händel and Haarmeier, 2009). This coupling is also recognized in the conscious access to visual target representation (Nakatani et al., 2014). As skill ability largely depends on visual discrimination, which is also dependent on the cerebellar projections onto the PFC and parietal cortex (Händel et al., 2009), a specific attention should be directed to delta-gamma coupling of the brain rhythm during sport activity. In this context, it is crucial to rule out contamination of EEG signals by EMG activity, movement artifacts, including from physiological activities (Ito et al., 2014; Lechinger et al., 2015). As mentioned above, the phase of delta oscillation plays important role when it facilitates MUA. It is also involved in predictive timing, explaining why the human beat processing is better when the stimulus tempo is in the delta range (1–3 Hz) (Cirelli et al., 2014).

## Theta oscillation

The medial septum-diagonal band of Broca (MS/DBB) reciprocally connected to the supramamillary nucleus plays the role of “theta pacemaker” for the hippocampal theta oscillation (Alonso et al., 1987; Vertes and Kocsis, 1997). It is modulated by different brainstem regions, including the nucleus incertus (NI), located in the dorsal tegmentum. The convergence of experimental observations about the NI and theta oscillation underlines the interest for future investigations of EEG theta in movement performance: (1) the electrical stimulation of the NI evoked theta oscillation in the rat hippocampus (Nuñez et al., 2006); (2) the NI neurons projecting to the cerebellar flocculus discharge in a burst-tonic mode in perfect correlation with the velocity and position of the eye in the cat (Cheron et al., 1995); (3) the theta hippocampal oscillation is synchronized with that in the cerebellum during eye-blink trace conditioning in the rabbit (Hoffmann and Berry, 2009; Wikgren et al., 2010). For all of these reasons future investigations of theta oscillation during head-eye movement in sports should be promising.

A clear theta peak at ~6 Hz has been recorded in hippocampal intracranial EEG (iEEG) in human (Kahana et al., 1999; Figures 5C–E). More recently, a “slow-theta” (~3 Hz) (memory related) and a “fast-theta” (~8 Hz) oscillation (not memory related) were defined (Lega et al., 2012, 2014; Pastötter and Bäuml, 2014).

### Phase-amplitude theta-gamma coupling

Theta oscillations enforce the timing of coordinated firing of neuronal populations, (Buzsáki, 2002; Buzsáki and Chrobak, 2005; Buzsáki and Watson, 2012). Recently, intracranial recordings in human demonstrated that hippocampal and neocortex phase amplitude coupling (PAC) exerted by slow-theta oscillation, respectively at 2.5–5 and 4–9 Hz modulates gamma activity. As episodic memory contains different types of information (semantic, spatial, emotional, and context dependence), it was suggested that the reinstatement of a specific context (integrating brain state dynamics and external elements) expressed into specific neuronal oscillations and spiking was at the basis of a novel model of episodic memory called “SCERT” (spectro-contextual encoding and retrieval theory) (Watrous and Ekstrom, 2014). This theory might offer testable predictions about the possibility to reinstate an episodic memory of a sport event by using VR realistic scenario and or motor imagery able to reproduce the cross-frequency coupling (e.g., delta-gamma or theta-gamma) and phase-synchronization originally present in the hippocampal-neocortical neuronal assemblies during mnemonic formation.

### Theta oscillation and speed cells

The dynamic aspect of self-representation in speed running is a crucial element for keeping the good trajectory for the best record. The recent discovery of the “speed cells” in the medial entorhinal cortex in the rat providing linear information to the grid cells (Hafting et al., 2005; Moser et al., 2008) represent a key element of dynamics representation of self-body location (Kropff et al., 2015). This discovery corroborates the fact that delta and theta oscillations recorded in human hippocampus (Watrous et al., 2011) during VR navigation increased their power not only as a function of spatial view (relevant goals and or spatial updating as initially demonstrated by for theta oscillation by Kahana et al., 1999) but also as a function of the speed of the VR image). This is also supported by the subdural grid and intracerebral microelectrode recordings in epileptic patients using a virtual taxi driver task (Caplan et al., 2003). These authors propose that the cortical theta reflects two functions depending of the behavioral context: (1) during goal-seeking, theta oscillation updates the motor plans in function of the incoming sensory information related to wayfinding; (2) during exploratory searching behavior, theta oscillation helps the encoding of coordinated activity in the multiple brain areas involved in building the individual's cognitive map. Theta rhythm also contributes to encoding the “where” and “when” of episodic memory (see Hasselmo and Stern, 2014, for a review).

### Theta in motor control

Theta oscillation plays also an important function in motor control. Recently, it was demonstrated that the theta power increase (ERS) in the contralateral motor area during the onset of fast ballistic movements (Ofori et al., 2015). Moreover, theta oscillation was phase-locked with the onset of the movement and the theta power correlated with movement acceleration (Ofori et al., 2015). A better performance in expert golfers was reported to be associated with higher fronto-midline EEG theta (FM theta) power and higher parietal upper alpha with respect to novices (Baumeister et al., 2008). In the same vein, Doppelmayr et al. (2008) showed a FM theta power increase for the last 3 s before the riffle shooting only in experts, compared to novices. Inverse modeling (LORETA) revealed theta source in the medial frontal cortex and anterior cingulate area underlying focused attention. Finally, (Bailey et al., 2008) reported an increase in theta activity above resting levels at higher workloads and at fatigue during graded cycle exercise.

## The alpha oscillation

**Figure 6:**
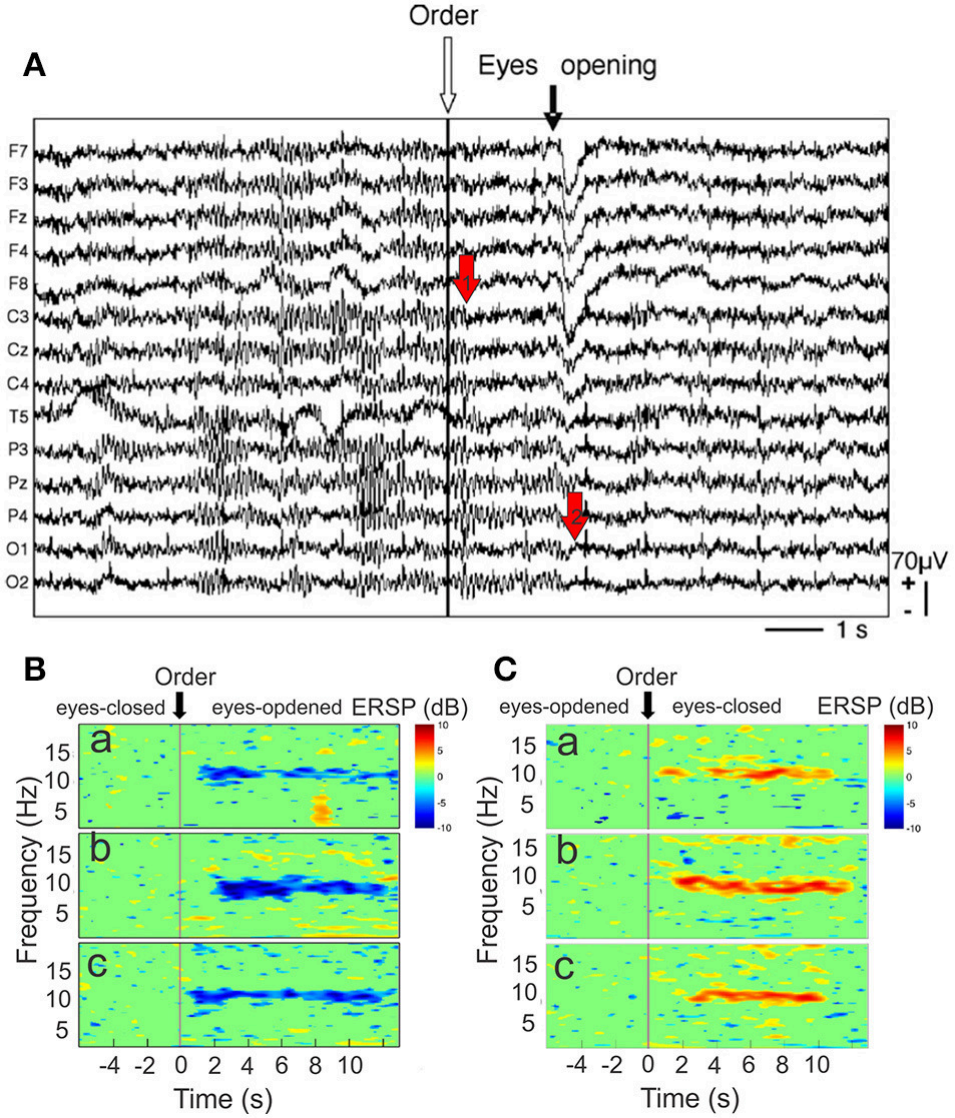
**(A)** Raw EEG recordings during the arrest reaction. Fourteen EEG channels referenced to linked mastoid (from F7 top) to O2 (bottom). The white arrow indexes to the order to open the eyes. The black arrow points to the onset of the eye movement artifact related to eye opening, mainly recorded by frontal electrodes (F7, F3, FZ, F4, F8). Note that the amplitude of mu rhythm [recorded by central electrodes (C3, CZ, C4)] is reduced before eye opening (red arrow “1”) whereas alpha rhythm (P3, P4, PZ, O1, O2) is only reduced after this movement when the eyes are closed. Event-related spectral perturbation (ERSP) time-locked to the order of eyes opening. **(B)** ERSP-image plot of the color-coded single trials of the EEG spectrum recorded in P3 channel in one cosmonaut on Earth before the flight **(a)**, in weightlessness **(b)**, and on Earth after the flight **(c)**. The trials are time-locked to the order to open the eyes (stripped line). Blue color indicates a decrease in power. **(C)** ERSP-image plot for the same electrode, condition and participant as in **(B)**, but time-locked to the order of eyes closing. Red color indicates an increase in power (modified from Cheron et al., 2006, with permission).

As the first recorded EEG wave (Berger, 1929), the alpha rhythm mainly appears as spindle-shaped episodes of 10-Hz oscillation that dominate the spontaneous activity of the brain in the relaxed eyes-closed state. When the eyes open the power of the alpha oscillation decreases (arrest reaction) (Figure 6) whereas faster oscillations with smaller amplitude occurred. The transition between these two eye states (closed vs. open) may provide an interesting index of alpha rhythm reactivity and may help to give direct information to the trainer about the global resting state activity of the athlete brain. For this we have defined a *suppression coefficient* (SC) as the peak value resulting from the subtraction of the power spectrum recorded in the eyes-closed state from that recorded in the eyes-opened state (Cheron et al., 2006).

The large amplitude of the alpha rhythm would result from a coherent cortical drive from the thalamus coincident with a lack of other sensory input. In this theoretical framework, that is compatible with Crick's searchlight hypothesis (Crick, 1984), a relaxed but well-focused athlete would demonstrate large alpha amplitude before the accomplishment of the motor task. The alpha rhythm has also been considered as a mechanism for increasing signal-to-noise ratios within the cerebral cortex by means of inhibition of unnecessary or conflicting processes to the current task (Klimesch, 1999; Klimesch et al., 2000; Cheron et al., 2006; Sadaghiani et al., 2012). In sport, the perturbation of the reference frame due to the modification of the environmental field and the sensory conflicts which could be produced by a new competitive field associate to psychological pressure could necessitate such a type of regulation.

Generated by interplay of the cortex and the thalamus and modulated by frontocorticothalamic loops, involving the reticular formation, and the pulvinar nucleus (Saalmann and Kastner, 2009, 2011) the 10 Hz oscillations are in fact composed of different rhythms depending of the sensorimotor modalities: the alpha oscillations of the visual system, the mu oscillations of the sensorimotor system (Gastaut, 1952; Arroyo et al., 1993; Ai and Ro, 2014) and tau oscillations of the auditory system (Lehtelä et al., 1997; Walz et al., 2015). ERD of mu rhythm also occurred when individuals (in an identification perspective with the subject on screen) observed movies showing biological motion (e.g., boxing) (Gastaut and Bert, 1954). Mu rhythm is maximally expressed over the sensorimotor areas during a relaxed state (Pfurtscheller and Neuper, 1994; Pfurtscheller et al., 1997) and reduced just before, during movement execution, tactile stimulation and motor imagery a condition interpreted as ERD (Figure 6B) in contrast to an amplitude enhancement referred to ERS (Figure 6C). Interestingly, the association of anodal transcranial direct current stimulation (tDCS) and motor imagery, two conditions known to increase the cortical excitability of the motor cortex (Nitsche and Paulus, 2001; Facchini et al., 2002, respectively), increase the ERD amplitude during motor imagery (Cebolla et al., 2015) in particular at the level of dominant hand (Kasuga et al., 2015). As motor imagery is more and more used in motor skill training (Schuster et al., 2011), the conjunction of tDCS, which is able to induces motor imagery (Speth et al., 2015), VR stimulation and motor imagery controlled by mu oscillation recording should be the future avenue for increasing CNS performance.

### The neural efficiency hypothesis

In line with the “neural efficiency” hypothesis suggesting that individuals with higher intelligent quotient (IQ) have lower brain activation than less bright individuals when functioning on the same cognitive tasks (Dunst et al., 2014), the same hypothesis has been extended to expert and athlete performances (Del Percio et al., 2009b, 2010, 2011; Babiloni et al., 2010). Based on the existence of an inverse relationship between IQ and the amount of upper alpha ERD (Grabner et al., 2004), it was proposed to use the classical “arrest reaction” to eye openings to quantify the alpha ERD amplitude in different participant groups. Namely, it was shown that elite karate athletes' brain is characterized by less ERD at the “arrest reaction” with respect to non-athlete participants (Del Percio et al., 2011). This corroborate previous experiments of the same group showing that elite athletes presented less alpha ERD during air pistol shoot than non-athletes (Del Percio et al., 2009a). However, the generalization of the alpha ERD must be cautiously made and greatly depends on the experimental condition. Indeed, during a body–sway paradigm the athletes have demonstrated a greater alpha ERD reflecting a better balance control than non-athlete (Del Percio et al., 2007).

### Alpha oscillation in binding or gating

Alpha oscillation is now increasingly regarded as reflecting a global inhibition of the cortex in order to exert a cognitive control of the final performance (Klimesch et al., 2007; Haegens et al., 2010). In this context, two seemingly opposite functions are operational. In the first function, the synchronization of alpha oscillations appears to integrate the multisensory factors into segregated chunks of information necessary for the binding of the inter-personal action perception loops. For example, Babiloni et al. (2008) reported that in golfers, the amplitude of the 10–12 Hz frequency alpha ERD was higher during the successful compared to the unsuccessful putts over the frontal midline (Fz and Cz electrodes), this increase reflecting fine motor control and balance abilities. In contrast, Taliep and John (2014) showed a significantly greater high-alpha (10–12 Hz) ERS prior to ball release. In the second function, the increase in alpha power may exert a “windshield wiper” effect through pulsed inhibition in order to select or gate the incoming signals (Sadaghiani et al., 2012). For example, in golfers, increased alpha activity in the left hemisphere was associated, and able to predict less error in the subsequent golf putt (Crews and Landers, 1993). This is consistent with subsequent finding of an increased alpha power for the best compared to worst shots in elite archers (Salazar et al., 1990). On the contrary, (Hillman et al., 2000) found an increase in alpha across both cerebral hemispheres during the preparatory period preceding shots for rejected (i.e., that were finally not accomplished), compared to executed shots. In the same vein, Dekker et al. (2014) demonstrated that feedback on alpha power (eyes open) based on music training in gymnast produce small improvements in sleep quality, mental, and physical shape. In absence of heart rate deceleration, alpha power was found to be greater in the left than in the right hemisphere during the period preceding the shot in elite archers (Salazar et al., 1990). In addition, this contralateral alpha power was greater for the best than the worst shots.

### Alpha oscillation phase

Another important function of alpha oscillation is made by its ongoing phase. By selecting the EEG trials that coincide with the P1 component generation of the visual evoked potential related to a recognition task, Gruber et al. (2014) showed that trials presenting alpha phase in the same polarity that the P1 component were associated with shorter target decision times than the rejected trials presenting counter phases to the P1. These results paves the way for precise checking of alpha phase oscillations during sport performance and direct BCI procedure based on instantaneous oscillating phases.

### Alpha and physical exercise

Many studies showed an association between different stages of exercising and modulations of the alpha rhythm. The findings are however very inconsistent. While the transition from rest to exercise has been linked to an increase in alpha (Nielsen et al., 2001; Rasmussen et al., 2004), the during and after exercise periods have been associated with both upward (Mechau et al., 1998; Rasmussen et al., 2004; Bailey et al., 2008; Schneider et al., 2009; Fumoto et al., 2010; Brümmer et al., 2011; Gutmann et al., 2015; Robertson and Marino, 2015; Mierau et al., 2016) and downward variations (Kubitz and Mott, 1996; Mechau et al., 1998; Doppelmayr et al., 2008, 2012; Schneider et al., 2009; Ludyga et al., 2015). Faced to apparently discrepant results about alpha rhythm behaviors related to exercise, and the existence of complex variation of the resting state EEG dynamics (Goldberg et al., 2008), we would suggest using better controlled paradigms linking alpha oscillation and specific sensory, cognitive, or motor triggering events.

## Beta oscillation

### Beta oscillation in motor control

Beta oscillation (15–30 Hz) occupies a central position in the treatment of sensorimotor information and serves as a functional link between different brain regions recorded in the monkey such as the pre-motor (Lebedev and Wise, 2000) motor (M1) (Donoghue et al., 1998; Reimer and Hatsopoulos, 2010) and somatosensory (S1) cortex (Lebedev and Nelson, 1995; Murthy and Fetz, 1996) the supplementary motor area (Hosaka et al., 2015) and the cerebellum (Courtemanche et al., 2002; Courtemanche and Lamarre, 2005). In these regions, beta LFP oscillations have been recorded in quietly sitting normal animals when they are immobile in expectancy and are inhibited by passive or active movements (Courtemanche and Lamarre, 2005). In the cerebellum, beta oscillations were localized in the granular layer where they are synchronized along the rostro-caudal module serving as a coordinated timing template (D'Angelo and De Zeeuw, 2009; Cheron et al., 2015). This oscillation is thus involved in movement preparation during which the Purkinje cell (PC) discharges (the sole output of the cerebellar cortex) is mainly phase-locked to this oscillation just before the execution of movement (Courtemanche and Lamarre, 2005). The phase relationship between beta oscillation, PC firing and movement execution is crucial.

By this way, beta oscillation could play the role of a motor “binding” linking the different commands related to a global gesture as suggested for sensory awareness (Engel and Singer, 2001). In the context of sport competition when exercise-induced-muscle-damage it was reported that the beta oscillation increase in relation to a force output decrease, neurosmuscular alteration and fatigue (Plattner et al., 2014).

In human cortex, Kuo et al. (2014) showed that beta-band (14–30 Hz) ERD (power decreases) was significant during finger movement, expressed in all participants and consistently localized to the primary motor cortex (hand region) and in well-accordance with fMRI localizations. As described for the mu rhythm, beta ERD also occur during both movement execution (Ofori et al., 2015) and observation (Zarka et al., 2014; Cevallos et al., 2015; Babiloni et al., 2016). This evidence was recently reinforced by LFP recordings in the monkey showing modulation of beta power in M1 and premotor cortex during action execution and observation (Waldert et al., 2015).

### Beta oscillation in sensory functions

In addition to sensorimotor area, beta oscillations play other important functions in pure sensory domains. For example, prominent increase in beta power (14–16 Hz) was reported for contour compared to non-contour conditions during a visual contour detection task (Volberg and Greenlee, 2014).

Beta oscillations were also modulated by somatosensory input (Pfurtscheller et al., 2001). More recently, in a new paradigm (Cebolla et al., 2014) based on the amplitude increase of the N30 component of the somatosensory evoked potential (SEP) during the observation of another person's hand movement (Rossi et al., 2002), we have demonstrated that beta ERS in a large central-posterior area extending from the contralateral motor cortex through the secondary somatosensory cortex to the insular and in the occipital secondary visual cortex participated to the N30 amplitude increase. In addition, beta phase-locking (ITC) was found in a large region including the premotor, motor, and somatosensory cortex and the angular gyrus (BA39) considered as an important node in the mirror neuron network (MNN) (Cebolla et al., 2014). These results demonstrate that beta oscillation participates in the top-down influence exerted by the MNN on the early evoked SEP component (Cebolla and Cheron, 2015).

### Beta oscillation and automatic nervous system

Recently, the low-frequency beta oscillations recorded in the Rolandic rhythms were demonstrated to be related to heart rate regulation by sympathetic activity (Triggiani et al., 2015). Beta oscillation can be thus viewed as a dynamic biomarker involving somatomotor control and ANS signals regulation.

The fact that contrasted results were obtained about the relationship between heart rate deceleration and performance accuracy (Konttinen et al., 1998; Tremayne and Barry, 2001) calls for direct investigation of the EEG rhythms during the preparatory phase of a movement skill. Interestingly, by linking motor imagery of a brisk movement of the leg and the go/no-go paradigm, Pfurtscheller et al. (2013) demonstrated that beta ERD was stronger for the go imagery than in the no-go imagery condition and that the heart rate deceleration was smaller and followed by an acceleration in go as compared to no-go. These authors interpret that this beta ERD increase was due to the increased mental effort linked to the imagery process.

## The gamma oscillation

Following Steriade (2003) the first description of gamma oscillation was made by Bremer et al. (1960) and described as an “*accélération synchronisatrice*” of the cortical EEG when the brainstem reticular formation was electrically stimulated.

### Binding-by-gamma oscillation

The first demonstration of the functional role of neuronal gamma-band synchronization was provided by invasive microelectrode recordings of spikes and LFP in the visual cortex of anesthetized cats (Eckhorn et al., 1988; Gray et al., 1989; Gray and Singer, 1989; Engel et al., 1991) and later confirmed in visual cortex of alert monkey (Kreiter and Singer, 1996; Fries et al., 2001). These important experiments have paved the way for the binding-by-gamma oscillation originally promoted by Freeman (1978) in the olfactory bulb. Novel signal-processing techniques for the detection of transient oscillatory events and the introduction of new source modeling methods (Lachaux et al., 2003; Delorme and Makeig, 2004; Palmero-Soler et al., 2007; Cebolla et al., 2011; Delorme et al., 2015) have facilitated the study of gamma oscillations in human EEG (see Jensen et al., 2007, for a review).

Since pioneering MEG studies (Ribary et al., 1991; Llinás and Ribary, 1993), the interest in the study of gamma oscillation with EEG and MEG increased strongly and their involvement was described in relation to sensorimotor task (Murthy and Fetz, 1992; Donoghue et al., 1998; Aoki et al., 1999), perception (Tallon-Baudry et al., 1996, 1997, 2005; Gruber et al., 2002), attention (Gruber et al., 1999; Gruber and Müller, 2006), working memory (Tallon-Baudry et al., 1998; Lutzenberger et al., 2002) and associative memory (Gruber et al., 2001). In gamma EEG recordings, Yuval-Greenberg et al. (2008) have demonstrated that the induced gamma-band EEG response occurring about 200–300 ms following stimulus onset correspond to miniature saccade dynamics rather than neuronal oscillations. However, this study does not raise any doubt regarding the intracranial recordings and the important role of gamma-band activity in neural function, but it urgently demonstrated the necessity to control not only the concomitant EMG and eye saccades but also the microsaccades events (Keren et al., 2010; Hassler et al., 2011, 2013) during any type of sport behaviors.

## Adaptation of oscillatory models to sports performance

**Figure 7:**
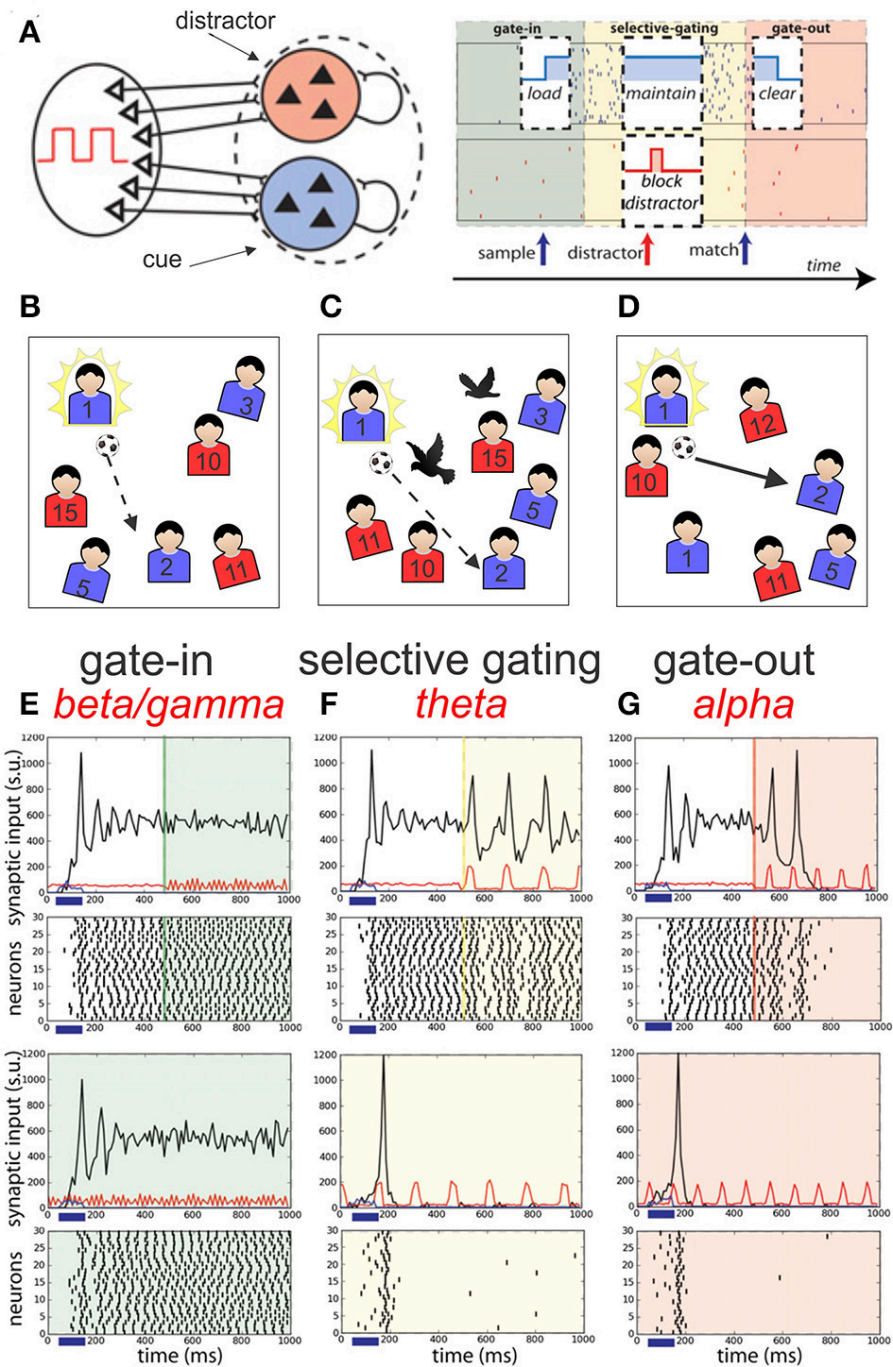
**Schematic representation of the network models of Dipoppa and Gutkin (2013) dedicated to a working memory task**. **(A)** Two neuronal populations B (in blue) and R (in red) of recurrent network receiving input by sources modulated by the shared background oscillation. The phases of operations are outlined in a white box showing the succession of the gating modes and operations: (I) Gate-in mode: the sample stimulus (blue arrow) activates population B (load). (II) Selective-gating: the distractor stimulus (red arrow) is not able to activate population R persistently (block distractor) and the memory in population B is held (maintain). (III) Gate-out mode: upon match-stimulus presentation, persistent activity is deactivated in the blue population (clear). Input oscillations enabling the gating modes: beta–gamma band ensures the gate-in mode at the beginning of the task, theta band ensures the selective-gating mode during the delay period (memory maintenance together with protections from the distractors), and alpha band ensures the gate-out mode at the task completion (memory is rapidly cleared). **(B–D)** Adaptive suggestion of this model into sport context (passing ball in soccer) (see main text for more details). **(E–G)** Frequency-dependent influence of oscillations on the reverberant spiking persistent state. The network responses (black) to a transient excitatory external stimulus (*t* = 50–150 ms) (blue bar and signal) depend on the frequency content of the background oscillatory input. First and third rows show the raster histogram of 30 representative neurons; second and fourth rows show average population input from recurrent connections (black), background activity (red), and external stimulus (blue) in arbitrary/normalized units. For each frequency the background oscillation is switched on either after the stimulus presentation (first and second rows) or before (third and fourth rows). **(E)** Beta–gamma-band oscillations (45 Hz) are compatible with persistent state maintenance: neither erasing nor blocking is seen. **(F)** Theta-band oscillations (6.5 Hz) maintain an a priori persistent state while blocking *de novo* activations. **(G)** Alpha-band oscillations (here 10 Hz) inhibit reverberant activity: the persistent state is deactivated by oscillations onset and is prevented from being activated by the transient stimulus (adapted from Dipoppa and Gutkin, 2013, with permission).

The delayed match to sample (DMS) with distractors task (Miller et al., 1991) has been used for testing the accuracy of a working memory (WM) model based on recurrently coupled networks of spiking neurons (Dipoppa and Gutkin, 2013; Figure 7A). A first visual sample cue is presented to the subject, after a delay period during which a random number of distractor cues are presented, the subject must only respond when the test cue matches the sample cue. This “go, no-go” WM task can be easily transposed in sport competition (starting for speed running or passing ball in soccer). In this later example (Figure 7B), the first visual cue can be represented by the presence in one part of the soccer field of a targeted teammate (n°2 in Figure 7B, *gate-in*), all the rest of non-targeted teammates corresponding to “no-go signals” already moving during a delay period during which distractor cues may occurred (black birds) (Figure 7C, *selective gating*). Only when the targeted teammate is recognized in a profitable situation the pass must be accomplished (Figure 7D, *gate-out*).

Dipoppa and Gutkin (2013) realized a computational framework that mechanistically links the four main oscillatory bands (alpha, beta, gamma, and theta) into a working memory (WM) network. In this model the coexistence of a ground state and persistent activity is allowed by the beta-gamma oscillation in such a way that transient input can initiate a persistent state corresponding to the *gate-in* mode of the dynamic gating regime (Figures 7E–G). During this mode the sample or targeted cue is acquired by the network. Then, the theta-band oscillations (3–8 Hz) assume a *selective-gating* mode maintaining in memory the previously loaded cue, assuming also a protection against distractors. Finally, a *gate-out* mode is provided by alpha-band oscillations (8–12 Hz) avoiding loading or maintenance of memory facilitating the memory cleaning and task completion.

This model includes two stimulus-selective neural population units (R representing red or B for blue) that are independently activated from a resting state to a persistent state by a sensory stimulus (Figure 7A). Although rather complex this model allows a better understanding of the dynamics links between persistent activities, transient input activation and the influence of different frequency band oscillations introduced in the network. For example, the Figures 7E–G shows that a transient excitatory input given between 50 and 150 ms (blue horizontal bar) on the reverberating network of neurons increases the synaptic activities (black line) showing multiple decreasing peaks reaching a relatively stable regime. Then if a gamma oscillation is added in the network (see green line at 500 ms) the stable level of the acquired synaptic activities is maintained (Figure 7E). The presence of gamma oscillation (45 Hz) at the time arrival of the external excitatory signal is ineffective. If theta oscillation (6.5 Hz) is given (yellow line) in place of the gamma one (Figure 7F), the mean level of activities is maintained but a peak of activities is reactivated at each theta wave. In contrast, if alpha oscillation (10 Hz) is added in the network (red line) (Figure 7G) after two initial reactivation peaks the excitatory level goes to zero. In addition, if theta or alpha oscillations are present at the time arrival of the external input no stable level of excitation are attained.

## Event related potentials in sport domain

As the majority of EEG studies devoted to sports have been included in the previous description of the different EEG rhythms, a review of the event related potentials (ERP) studies in sport is accomplished in the last part of the present review. Independently of the analysis of EEG content *per se*, two types of ERP studies have been conducted, one about the effect of chronic physical activity and the other about the effect of acute physical exercise.

### Effects of chronic physical exercise

The first set, which makes up the majority of the studies, focused on the relationship between neuroelectric modulations associated with cognitive functioning and engagement in chronic physical activity through the life span. Studies using tasks involving stimulus discrimination and attention processes found that chronic engagement in exercise and aerobic fitness are linked to higher capacities, as reflected for the most part by larger P3 amplitudes, themselves indexing increased attentional resources during stimulus encoding (Hamon and Seri, 1989; Polich and Lardon, 1997; Hillman et al., 2005; Pontifex et al., 2009; Chang et al., 2012; Taddei et al., 2012; Getzmann et al., 2013), and by shorter P3 latencies, marking greater cognitive processing speed (Dustman et al., 1990; Hillman et al., 2002, 2004, 2005; Chang et al., 2012; Taddei et al., 2012). These ERP modulations correlated with reduced reaction times and greater response accuracy in the tasks proposed. Only one study found opposite results, i.e., a more efficient capacity in perceptual decision making in baseball batters associated with longer P3 latency and reduced P3 amplitude (Radlo et al., 2001). Studies using the same type of tasks but which have looked into the effects of physical activity on pre-stimulus stages of motor preparation and very early stages of stimulus processing have also reported modulations of earlier components such as greater N1, (Hamon and Seri, 1989; Hung et al., 2004; Berchicci et al., 2015), P1, (Berchicci et al., 2015), P2 (Hamon and Seri, 1989), or N2 amplitudes (Taddei et al., 2012; Winneke et al., 2012), and shorter N2 latency (Taddei et al., 2012). Despite the difference in the tasks used, the majority of authors having investigated the ERP correlates of inhibition (Kamijo and Takeda, 2009; Stroth et al., 2009; Hillman et al., 2014; Chu et al., 2015; Gajewski and Falkenstein, 2015; Kamijo et al., 2015) have also reported increased P3 amplitudes. Greater amplitudes of CNV, indicating greater task preparation processes, as well as diminished N2 amplitudes, reflecting more efficient executive control processes, have also been reported (Stroth et al., 2009). These observations thus suggest a strong positive association between chronic physical activity and the performance in inhibition capacity.

Chronic exercise has also been found to be linked to smaller frontal CNV amplitude during the speed instruction in a Sternberg task (Kamijo et al., 2010, 2011), which indexes more efficient cognitive preparation for working memory. A facilitation of working memory in individuals practising physical activity has also been indexed by an increase in P3 (Chang et al., 2013; Dai et al., 2013) and N1 amplitudes (Chang et al., 2013) and a decrease in P3 latencies (Chang et al., 2013; Fong et al., 2014) in working memory paradigms, which suggests greater allocation and engagement of attentional resources and higher efficiency of stimulus evaluation. Numerous studies (Themanson et al., 2006; Themanson and Hillman, 2006; Hillman et al., 2009a; Pontifex et al., 2011) reported a relationship between higher chronic physical activity and reduced event related negativity (ERN) amplitude and greater post-error response slowing under speeded task instructions. These effects could be interpreted as a decrease in conflict monitoring or an inferior threshold for the moment at which the several processes necessary for the upregulation of cognitive control start to be activated.

Themanson et al. (2008) showed that fitness was associated with larger modulation of ERN amplitude and post-error accuracy crosswise the task instruction conditions during a task requiring variable cognitive demands and Pontifex et al. (2011), through manipulation of stimulus–response compatibility in a modified Flanker task, showed higher response accuracy, larger amplitudes and shorter latencies of the P3 and greater ERN amplitude modulation in higher aerobically fit participants. These two studies indicate increased cognitive flexibility of action monitoring in higher fit individuals. Hillman et al. (2006) and Kamijo and Takeda (2010) also found ERPs indices of higher cognitive flexibility in physically active individuals compared to sedentary ones. However, others researchers (Scisco et al., 2008) did not find any link between physical activity and cognitive performance in a task switching paradigm. This absence of association has been explained in difference in task difficulty of the tasks chosen (Hillman et al., 2012).

Finally, higher fitness appears to be linked to a richer network of words and their meanings, and a greater ability in detecting and correcting syntactic errors as indexed by the greater N400 amplitude and shorter latency and the larger P600 for syntactic violations during the reading of sentences (Scudder et al., 2014). It is important to note that the effects of physical activity on executive functioning-related ERPs are observed across all age groups. Higher levels of fitness indeed increase cognitive performance in adults (Themanson and Hillman, 2006; Bullock and Giesbrecht, 2014), but also in children (Drollette et al., 2014; Hillman et al., 2014) and may also help lightening cognitive decline with age (Hayes et al., 2013).

### Effects of acute physical exercise

The second set of researches has focused on studying the effect of acute physical activity (i.e., single bout of exercise lasting from a few seconds to several hours) on ERPs measurements. In this category, two types of work have been conducted. While some explored the effects of activity right following exercise, others studied the modulations of ERPs during exercise.

The studies that have concentrated on the after-effects of a single acute bout of exercise on neuroelectric indices of cognition have focused mainly on the ones reflecting inhibition (Kamijo et al., 2004, 2007, 2009; Hillman et al., 2009b, 2003; Drollette et al., 2014; Chuang et al., 2015; Chu et al., 2015; Kim et al., 2015) and stimulus inhibition discrimination processing (Nakamura et al., 1999; Yagi et al., 1999; Magnié et al., 2000; Kamijo et al., 2004; Duzova et al., 2005; Chang et al., 2012; Pontifex et al., 2015). A handful of studies also explored the correlates of action monitoring (Hillman et al., 2006; Chang et al., 2015) and attention related brain activity (Pontifex et al., 2013; Tsai et al., 2014; Chang et al., 2015).

Overall, these studies, across different cognitive paradigms, showed that exercising during 20–30 min usually lead to greater P3 amplitude and shorter P3 latency, thus reflecting respectively an intensification of the mobilization of attentional processes and an increase in speed of cognitive processing and stimulus classification. Moreover, these exercise-consecutive improvements in cognition abilities seem to be dependent of the intensity of the exercise. In particular, only moderate (in opposition to high or low) intensity exercise appears to be advantageous for ERPs components associated with cognition (Hillman et al., 2012).

The nature of the changes occurring in the brain during exercise has been very much less studied. As far as we know, and this is probably due to technical problems linked to artifacts associated with the gross motor movement intrinsic within physical activity, only four studies have acquired ERP data during acute exercise (Yagi et al., 1999; Grego et al., 2004; Pontifex and Hillman, 2007; Bullock et al., 2015) and different aspects of cognition have been approached by the different authors. Globally, modulations of the N1, P1, P2, and N2 components have been observed, suggesting a complex effect of exercise on the neural indices of information processing, but the small and disparate amount of literature available at this stage renders conclusions hard to be drawn today.

In summary, cumulating evidence demonstrates that higher engagement in physical activity and higher practice of aerobic fitness can have important sustained and transient benefits across the human lifespan. They are linked to modulations of ERPs which attest to increased cognitive functioning, especially as far as executive functions are concerned. Preliminary findings suggest that specific modulations also occur during physical activity participation but further examinations are needed in this domain.

## Toward EEG biomarkers in sport

The Tables 1, 2 are constructed on the Pubmed search targeting *EEG-sport and exercise* and *Evoked potentials-sport and exercise*, respectively. This dichotomy between EEG and evoked potentials studies in sport domain is not surprising and follows the previous distinction existing in clinical studies. The EEG search resulted in about 30 studies, while the evoked potentials resulted in about 55 studies, the earliest dating back from 1989. These relatively small amounts of studies dedicated to sports compared to the large total number of EEG and evoked potentials studies raises serious questioning with regard to the interest in sports in the scientific community. In an optimistic view, we are thus at the beginning of new explorative era for which the establishment of a sound, comprehensive methodology will be helpful to better understand the brain functioning in sport performance.

**Table 1:**
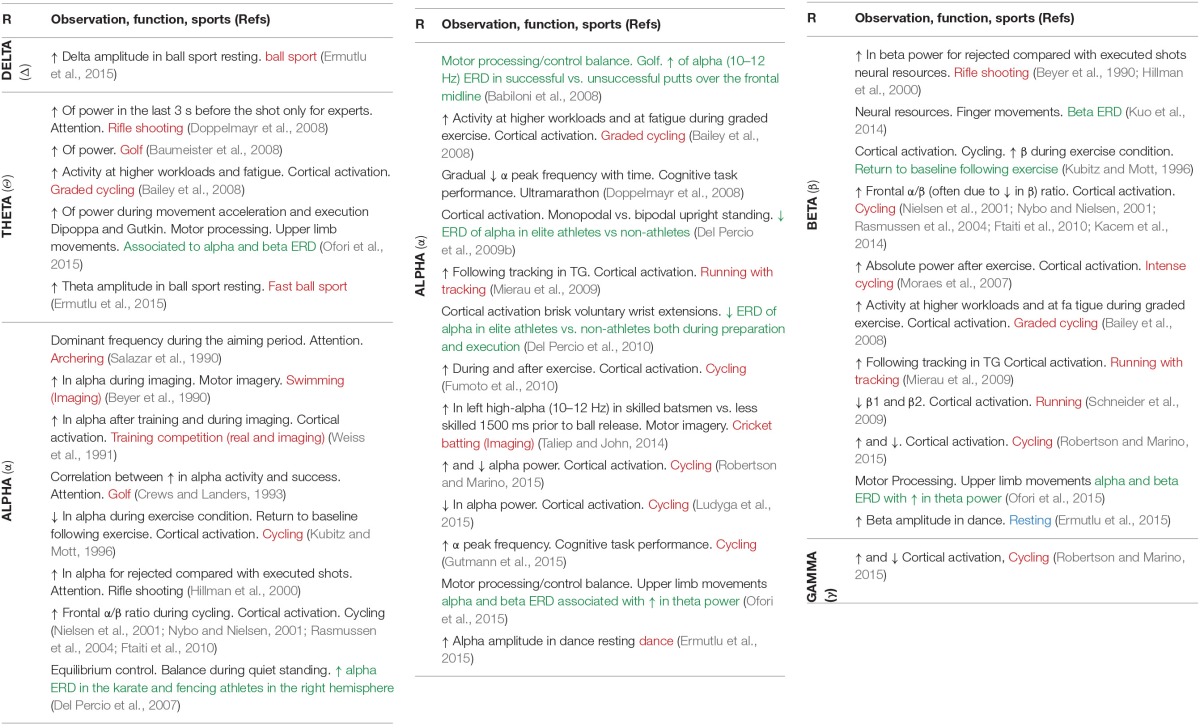
**EEG and physical activities**.

**Table 2:**
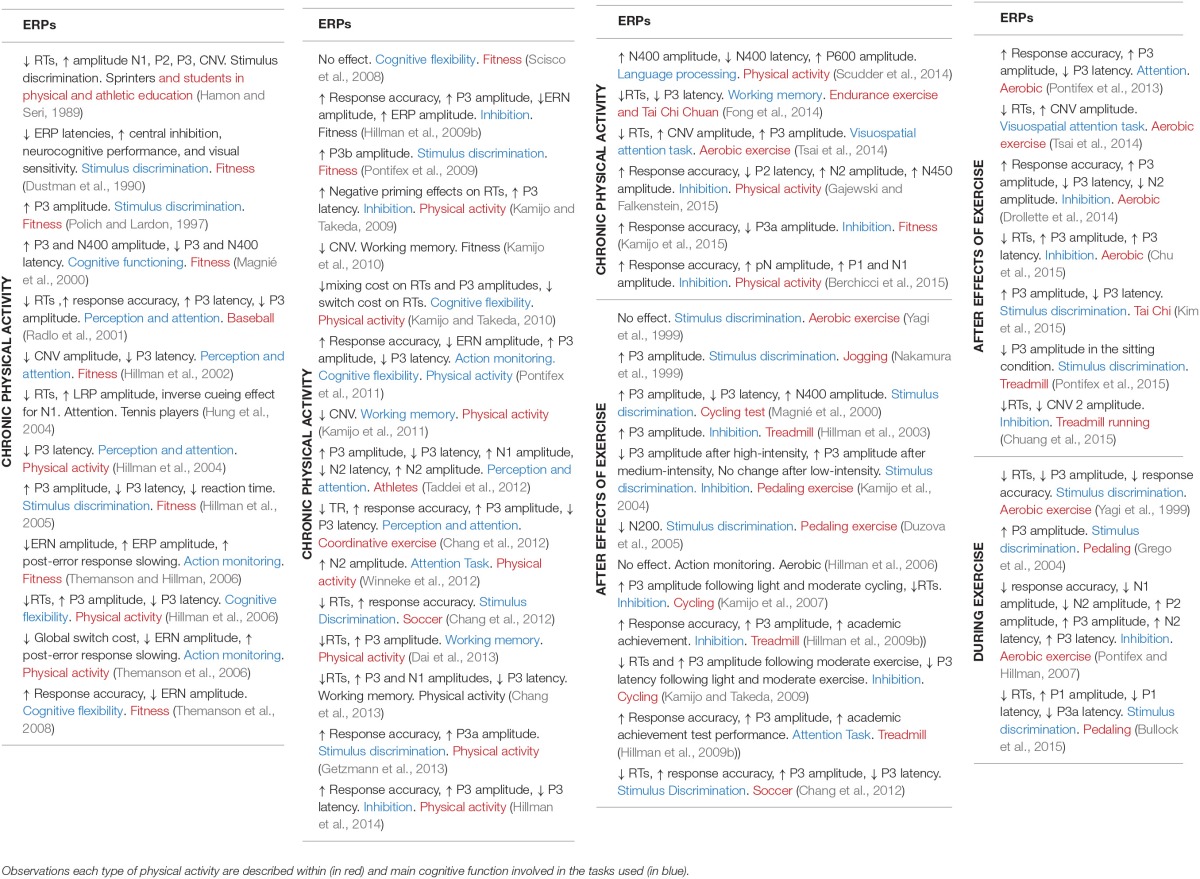
**Chronic, after, and during effects of physical activity on EEG evoked potentials (ERPs)**.

It also appears that the dynamics aspects of the EEG have only been recently envisaged, in 9 studies (green text in Table 1). The practical conclusions are not obvious at this stage because of the variability in the sports studied, paradigms used, analysis methods and interpretations. In spite of this situation and the few discordances (cited above, Table 2) the conjunction of physiological conclusions adopted thanks to the development of basic research realized (both in animals and human) outside of the sport domain and the more practical investigations may allow to attempt to define the basic line of EEG biomarkers in order to explore the brain activity associated to sports. We may summarize a perspective view about five major EEG biomarkers hierarchically classified following the number of corresponding functions (see Table 3). These biomarkers could be used following EEG dynamics method and practically applied in sport domain. In order to avoid some confusions and insufficiencies in the treatment of these EEG biomarkers we recommended the adoption of an integrated approach (see below).

**Table 3:**
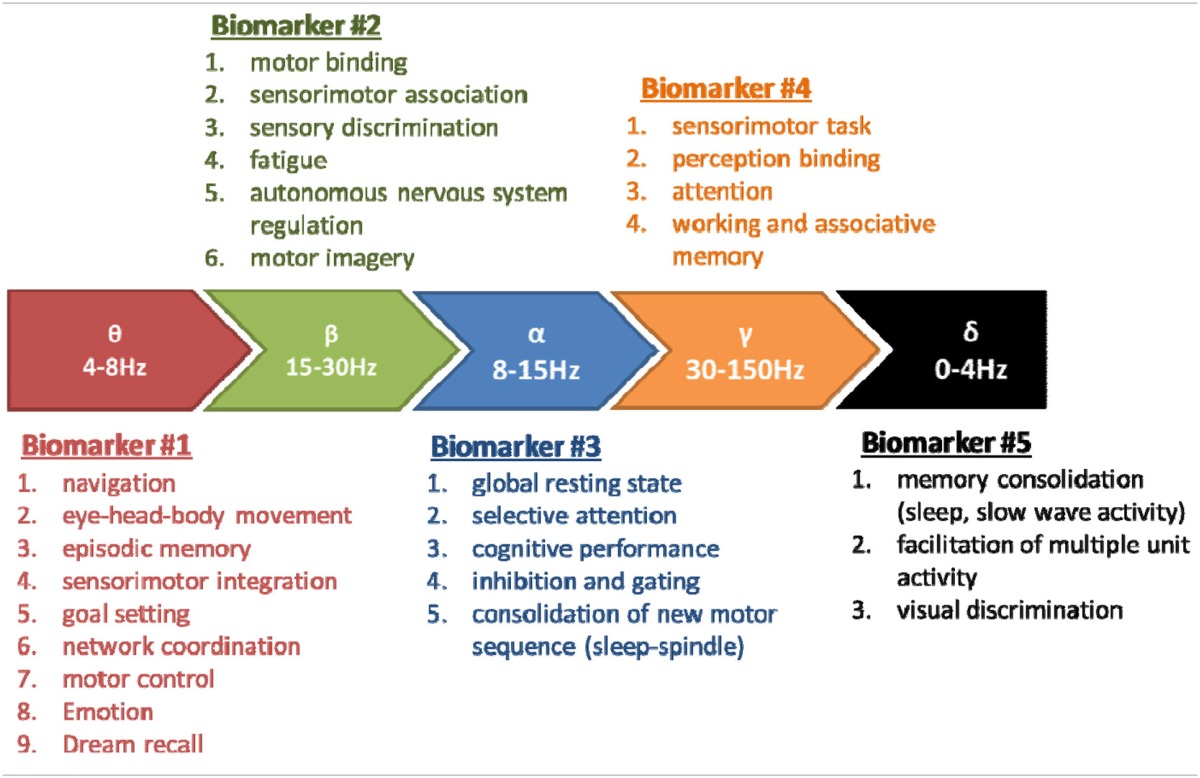
**Five EEG biomarkers hierarchically organized following the number of corresponding functions**.

## From walking studies to an integrated approach of brain activities in sport

The recording of EEG during normal or treadmill walking (Gwin et al., 2010, 2011; Cheron et al., 2012; Gramann et al., 2014a,b) has paved the way for further investigations in sport movement involving higher acceleration and shocks than the cyclic walking movement. Adopting the independent component analysis (ICA) method, already used in walking studies by Gwin et al. (2010, 2011) we have shown contralateral modulations at electrode locations C3–C4 of the mu-beta and gamma oscillations in relation to the respective heel strikes (Cheron et al., 2012). This confirms previous studies (Gwin et al., 2010, 2011; Haefeli et al., 2011) and is line with more recent investigations (Severens et al., 2012; Wagner et al., 2012; Seeber et al., 2014, 2015; Gramann et al., 2014b; Bruijn et al., 2015; García-Cossio et al., 2015; Lisi and Morimoto, 2015). In addition, we have shown that it is possible to use, after some artifact processing, the two most representative independent components of the EEG located over the sensorimotor cortex as input for a dynamic recurrent neural network (DRNN) learning identification of the two principal components (PCk1-2) of the three elevation angles (foot, shank, and thigh) of one lower limb kinematics (Cheron et al., 2012). This could motivate further application joining EEG dynamics and DRNN learning in sport domain. In spite of these advances and in accordance with the recent review of Gramann et al. (2014a) the main obstacle for a direct application of EEG to all the movement phases in sport remains movement artifacts. During treadmill walking the signals issued from EEG cap and accelerometer (placed on the subject's head) exhibit similar time-frequency properties, especially in frequency bands extending from the low-delta to high-gamma bands (1–150 Hz) (Castermans et al., 2014). This finding questioned some conclusions drawn in previous EEG studies where low-delta band (~1 Hz, the fundamental stepping frequency) had been interpreted as related to the control of lower limbs during locomotion. Concerning the gamma band oscillation, the contamination of EEG signals by cranial, facial and neck muscles activities occurring mainly between ~20 and 300 Hz represent another difficulty for which different solutions have been recently reviewed (Muthukumaraswamy, 2013) providing satisfactory options. In contrast to movement and EMG artifacts which are largely distributed throughout the scalp or specifically distributed laterally (close to neck, temporal and frontal region), respectively, the sensorimotor or cognition-related oscillations (e.g., mu and beta) change within narrow frequency bands and in more restricted locations.

Since the early EEG studies of treadmill walking, the EEGLAB tool-box (Delorme and Makeig, 2004) has been used in order to suppress different types of artifacts by the ICA. This has afforded significant progress in the EEG and evoked potentials domains, but the important problem of the movement artifacts occurring during the crucial phase of sport movement still requires new technological advances are. An original procedure described by Kline et al. (2015) consists of recording pure movement artifacts from a standard EEG headset placed on a silicone swim cap. Interestingly, the observed ERD/ERS from 1 to 150 Hz in the artifact data were similar to those obtained in gait-related studies using scalp EEG. Although the same group (Snyder et al., 2015) showed that the vast majority (99%) of the IC were located outside the brain and lacked dipolar characteristic (applying ICA on these pure artifact data), the authors concluded that “*Caution should be exercised when interpreting ICA for data that includes semi-periodic artifacts including artifact arising from human walking*.” In this context, a hardware system incorporated in each electrode should be developed in order to directly measure the actual movement artifact and reject (subtract) it from the real EEG signal. Another already developed option uses a miniaturized wireless EEG system composed of a ring of 8 electrodes embedded in flexprint biocompatible polyamide placed around the ears (Debener et al., 2015). This system seems to avoid electrode displacement, movement signal contamination, non-stationarities and impedance changes.

Some components of movement artifacts can also be relevant to fine analysis the brain control of movement. For example, blink (Wascher et al., 2014), eye movements including saccades (Cordones et al., 2013) and microsaccades (Yuval-Greenberg et al., 2008), or muscle twitches (Cohen and van Gaal, 2014) can be related to specific brain activities and inform about the current brain state. Rather than just ruling out the artifactual interference of these features on the EEG signals, they should be selectively extracted and explored with respect to preceding and subsequent EEG content.

In an optimistic view, we may therefore anticipate that the general problem of artifact produced by sport movements in the EEG signal will find reasonable solution in a very short time. This will greatly facilitate the generalization of integrated mobile platforms in sport domain as the one proposed by *MoBI* (mobile brain/body imaging) project for natural cognition (Gramann et al., 2014a,b). *MoBI* can synchronize multiple physiological parameters (motor, ocular, and autonomic) and sensori-physical environment. In this context, a new open source tool-box called MoBILAB (Ojeda et al., 2014) working with EEGLAB greatly help with the management and the treatment of the multiple streams of data.

Confronted to the large variety of intervening parameters in sport performance we propose a general working methodology which could be applied whatever the highly specialized sport disciplines. This future approach should be made within a technological platform assuming the perfect synchronization between (1) biomechanical recordings (3D movement, force), (2) multiple EMG, (3) high density wireless EEG, (4) eye movement, and (5) signals coming from the autonomous nervous system. Based on the initial feedback of the trainer's team, the targeted movement priorities will be defined and recorded in a first explorative way. The best performance of the best performers will be then compared to less good performance. Sensori-motor strategies based on the dynamic relationship between the multiple EMG profiles and the related kinematics or kinetics will be accurately identified and quantified with the help of DRNN technology (Cheron et al., 2012; Bengoetxea et al., 2014), and the EEG biomarkers of the related performance will be defined and quantified by mean of ERP analysis linked to external or internal movement events and ongoing high density EEG signals allowing further mathematical analysis such as time frequency (ERSP and ITC) (EEGLab procedures, (Delorme and Makeig, 2004; Delorme et al., 2015)) microstates analysis (CARTOOL) (Brunet et al., 2011), coherency/directionality (Nolte et al., 2004; Cheron et al., 2014) and inverse modeling of the ERP, ERS, ERD, and ITC neural generators (swLORETA) (Cebolla et al., 2011, 2014).

## Author contributions

Substantial contributions to the conception or design of the work (GC, GP, JC, AL, AC, CC, MP, TH, AC, and BD). Drafting the work or revising it critically for important intellectual content (GC, GP, GP, and TH). Final approval of the version to be published (GC, GP, BD, and TH). Agreement to be accountable for all aspects of the work in ensuring that questions related to the accuracy or integrity of any part of the work are appropriately investigated and resolved (GC and CC).

## Funding

We would like to thank T. D'Angelo, C. de Scoville, M. Dufief, E. Toussaint, E. Hortmanns, and M. Petieau, for expert technical assistance. This work was funded by the Belgian Federal Science Policy Office, the European Space Agency (AO-2004, 118), the Belgian National Fund for Scientific Research (FNRS), the research funds of the Université Libre de Bruxelles and of the Université de Mons (Belgium), the FEDER support (BIOFACT), the MINDWALKER project (“FP7” 2007-2013) supported by the European Commission, the Fonds G. Leibu and the NeuroAtt BIOWIN and Easy Move projects supported by Walloon Country of Belgium.

### Conflict of interest statement

The authors declare that the research was conducted in the absence of any commercial or financial relationships that could be construed as a potential conflict of interest.
